# Analysis of the efficacy and safety of endoscopic full-thickness resection in the treatment of rectal neuroendocrine tumors

**DOI:** 10.3389/fmed.2026.1816591

**Published:** 2026-05-20

**Authors:** Linzhen Li, Hui Yin, Asi He, Yayun Ye, Yan Zhang

**Affiliations:** 1 Department of Gastroenterology, The First Affiliated Hospital of Wannan Medical College, Wuhu, Anhui, China; 2 Department of Infection Management, The First Affiliated Hospital of Wannan Medical College, Wuhu, Anhui, China

**Keywords:** delayed bleeding, endoscopic full-thickness resection, endoscopic submucosal dissection, positive margins, rectal neuroendocrine tumors

## Abstract

**Background and purpose:**

Rectal neuroendocrine tumor (NET) is relatively common together with pancreatic NET in the context of a rare disease. Endoscopic resection is a commonly used treatment method. The aim of this prospective study is to analyze the safety and efficacy of endoscopic full-thickness resection (EFTR) in the treatment of rectal NET.

**Patients and methods:**

Patients who met the inclusion and exclusion criteria were randomly assigned to the endoscopic submucosal dissection (ESD) group and the EFTR group. Compare whether there are statistically significant differences between the two groups in terms of postoperative complications, hospital stay, and the rate of positive surgical margins.

**Results:**

This study included 58 patients, 43 patients were in the ESD group and 15 patients were in the EFTR group. There were no significant statistical differences between the two groups in terms of gender (*P* = 0.975), age (*P* = 0.477), and length of hospital stay (*P* = 0.207). Neither of the two groups of patients experienced delayed perforation after the operation (*P* = 1.000). In the ESD group, there was one patient who experienced delayed bleeding, while in the EFTR group, there were no patients with delayed bleeding (*P* = 0.746). In the ESD group, 4 patients had positive margins, while in the EFTR group, no patients had positive margins (*P* = 0.291). In the ESD group, 17 patients had tumor margins less than 500 micrometers from the bottom, while in the EFTR group, no patient had such a condition (*P* = 0.002).

**Conclusion:**

Endoscopic full-thickness resection combined with endoscopic purse-string suture is a safe method for treating rectal NETs, and it has a higher complete resection rate compared to ESD.

## 1 Introduction

Neuroendocrine tumors (NETs) are a type of rare tumors that originate from peptide-producing neurons and neuroendocrine cells, possessing neuroendocrine differentiation characteristics and expressing neuroendocrine markers. They can occur in various parts of the body, with the lungs and gastrointestinal tract being the most common sites (1). Among Asian populations, the pancreas and rectum are the most common sites of disease (1). A study showed that the incidence of rectal NETs is as high as 60% of gastrointestinal NETs in South Korea and Japan (2). Rectal NETs are mostly located in the middle and lower parts of the rectum, and are often solitary. Under white light endoscopy, their typical appearance is an isolated, round, broad-based or sessile submucosal protrusion. The mucosal surface is intact and smooth, presenting a pale yellow or pale color, and the texture is relatively hard (3, 4). According to the 2019 WHO Classification of Tumors of the Digestive System, well-differentiated neuroendocrine tumors can be classified as G1, G2 or G3, while poorly differentiated neuroendocrine carcinomas are defined as G3 (5, 6).

Neuroendocrine cells can produce, store and secrete various bioactive substances, thereby causing different clinical syndromes (7). Based on whether they secrete bioactive substances, they can be classified as functional or non-functional. Approximately 20%–30% of neuroendocrine tumors are functional tumors (8, 9). However, dysfunctional NETs can also cause related clinical symptoms, among which the most common symptom is abdominal pain (10, 11). Among them, the incidence of rectal NETs is on the rise globally (12, 13). Most rectal NETs have no symptoms and are discovered incidentally during colonoscopy. A few rectal NETs may present with hematochezia, anal discomfort, and changes in bowel habit (14, 15).

The treatment methods for NETs include surgical treatment, endoscopic treatment, drug therapy, interventional therapy, and radiation therapy. Since most rectal NETs are smaller than 10 mm and are of grade G1 or G2, most of them only require endoscopic treatment. The endoscopic treatment methods include m-endoscopic mucosal resection (m-EMR), endoscopic submucosal dissection (ESD), endoscopic full-thickness resection (EFTR), and endoscopic submucosal dissection between muscle layers (3). Among them, ESD is currently the most commonly used endoscopic method for treating rectal NETs. However, ESD also has some complications and cases of positive margins. A retrospective study showed that among 40 cases of rectal neuroendocrine tumors treated with ESD, 5 cases had positive margins, 2 cases experienced delayed bleeding, 1 case had delayed perforation, and 1 case had postoperative recurrence (16). Compared with ESD, the resection area of EFTR is larger, so the positive rate of the surgical margin should theoretically be lower than that of ESD. However, the wound area is also larger, and the postoperative wound management is also more complicated. A retrospective study published in 2020 (12) showed that 40 patients with rectal NETs underwent complete resection. The rate of full-layer resection was 95%, and no major adverse events occurred. However, at present, there are hardly any prospective studies comparing ESD and EFTR in the treatment of rectal neuroendocrine tumors. Therefore, the purpose of this prospective study is to compare the efficacy and complications of ESD and EFTR in the treatment of rectal NETs.

## 2 Materials and methods

### 2.1 Study design

The objective of this study was to compare the efficacy and complications of ESD and EFTR in the treatment of rectal NETs. The inclusion criteria were as follows: (1) patients with rectal NETs indicated by colonoscopy, (2) hospitalization was for endoscopic treatment. The exclusion criteria were as follows: (1) patients who have been clearly identified as requiring transfer, (2) the postoperative pathological result was not a neuroendocrine tumor, (3) extremely poor cardiac and pulmonary function, (4) patients with other active gastrointestinal diseases or synchronous second primary malignancies.

### 2.2 Patients and methods

**FIGURE 1:**
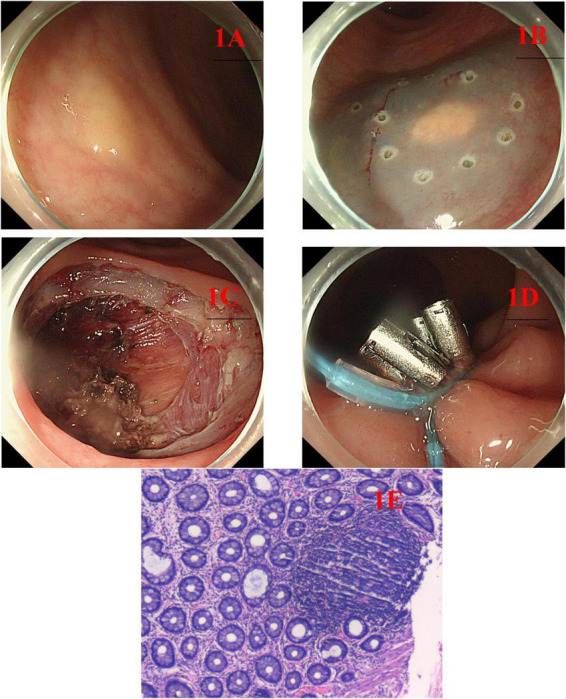
**(A)** A submucosal tumor 6 cm away from the anal margin, with a yellowish color; **(B)** mark at a distance of 0.5 cm from the tumor edge, and perform submucosal injection using the diluted indigo carmine solution; **(C)** perform endoscopic full-thickness resection of the tumor along the outer edge of the marked point; **(D)** closing the wound using the nylon suture combined with titanium clip purse suture technique; **(E)** the postoperative pathology indicated a neuroendocrine tumor.

A total of 58 rectal NETs patients (May 2023 - December 2025) in the department of gastroenterology were include in this study. On the first day of the patient’s admission, record their gender, age, diameter of the lesion and whether they have had hypertension, diabetes, chronic renal failure, or coronary heart disease. On the second day of admission, blood routine tests and coagulation function tests were conducted, and platelet counts and international standardization ratio (INR) were recorded. Patients were allocated alternately to the ESD or EFTR group on the basis of their recruitment sequence. Patients assigned to the EFTR group who did not agree to this endoscopic treatment method were then assigned to the ESD group. In the EFTR group, endoscopic purse-string sutures with titanium clips and endoloops were used to close the surgical wound (Figure 1). In the ESD group, only titanium clips were used for wound closure.

All patients underwent enhanced computed tomography (CT) of the entire abdomen preoperatively to rule out distant or lymph node metastasis and other conditions that are not suitable for endoscopic resection. All procedures were performed by the same physician with more than 5 years of experience in ESD treatment to avoid biases caused by different operators.

All patients were recorded for the occurrence of delayed bleeding and delayed perforation after the operation, as well as the postoperative pathological grade, whether the resection margin was positive, and whether there was vascular and nerve invasion. Delayed bleeding refers to bleeding that occurs within a few hours to 1 month after the operation. Then, we compared whether there were any statistically significant differences between the two groups in terms of the rates of delayed postoperative bleeding, delayed perforation, positive margins.

### 2.3 Ethical considerations

The research was performed according to the Declaration of Helsinki including patients’ consent. The study was approved by the institutional review board. All participants give informed consent.

### 2.4 Statistical analysis

Descriptive data are expressed in terms of median (interquartile) or percentage. All numerical variables were tested for normal distribution. Mann-whitney U test was used for nonparametric tests and Chi-square test or Fisher’s exact test was used for categorical variables. SPSS 21.0 software was used for statistical analysis. A *P*-value < 0.05 indicated statistical significance.

## 3 Results

### 3.1 Analysis of the basic characteristics between ESD group and EFTR group

This study included a total of 58 rectal NETs patients. Among them, there were 43 patients in the ESD group and 15 patients in the EFTR group. There were no significant statistical differences between the two groups in terms of gender (*P* = 0.975), age (*P* = 0.477), diabetes (*P* = 0.782), hypertension (*P* = 0.756), chronic kidney disease (*P* = 1.000), coronary heart disease (*P* = 0.470), and the use of antiplatelet or anticoagulant drugs (*P* = 0.608). Similarly, there were no significant statistical differences between the two groups in terms of INR (which reflects coagulation function) (*P* = 0.323) and platelets (which reflect hemostasis function) (*P* = 0.845) (Table 1).

**TABLE 1:** Analysis of the basic characteristics between ESD group and EFTR group.

Parameters	ESD group *n* = 43	EFTR group *n* = 15	*P*
Sex (M/F)	26/17	9/6	0.975
Age (years): M(QR)	50 (36–57)	50 (45–59)	0.477
Platelets: *n*, ×10^9^/L	196 (147–221)	193 (152–240)	0.845
INR: *n*	0.98 (0.95–1.02)	0.97 (0.94–1.00)	0.323
Diabetes: *n* (%)	1 (2.33%)	0 (0%)	0.782
Chronic kidney disease: *n* (%)	0 (0%)	0 (0%)	1.000
Hypertension: *n* (%)	14 (32.56%)	4 (26.67%)	0.756
Coronary heart disease: *n* (%)	3 (6.98%)	0 (0%)	0.470
Taking antiplatelet or anticoagulant drugs	2 (4.65%)	0 (0%)	0.608

M, median; QR, Quartile Range; INR, international normalized ratio; ESD, endoscopic submucosal dissection; EFTR, endoscopic full-thickness resection.

### 3.2 Comparison of postoperative complications and positive rates of margins between the two groups

There was no significant difference between the two groups in terms of diameter of the lesion (*P* = 0.070) and length of hospital stay (*P* = 0.207). The postoperative pathological results of the ESD group were all grade G1. In the EFTR group, the postoperative pathological results showed 14 cases of grade G1 and 1 case of grade G2. There were no patients with grade G3 in either group. Neither of the two groups of patients experienced delayed perforation after the operation (*P* = 1.000). In the ESD group, there was 1 patient who experienced delayed bleeding after the operation. In the EFTR group, no delayed bleeding occurred. However, the difference was not statistically significant (*P* = 0.746). In the ESD group, 4 patients had positive surgical margins after the operation. In the EFTR group, all surgical margins were negative after the operation, but the difference was not statistically significant (*P* = 0.291). There was one patient with vascular invasion in the ESD group, while no patient had vascular invasion in the EFTR group. The difference also was not statistically significant (*P* = 0.746). In the ESD group, 17 patients had tumor margins less than 500 micrometers from the bottom, while in the EFTR group, no patient had such a condition. The difference was statistically significant (*P* = 0.002) (Table 2).

**TABLE 2:** Analysis of postoperative complications between ESD group and EFTR group.

Parameters	All participants *n* = 58	ESD group *n* = 43	EFTR group *n* = 15	*P*
Diameter of the lesion: M(QR), mm	8 (5–10)	8 (6–10)	6 (5–8)	0.070
Length of hospital stay: *n*	7 (7–9)	8 (7–9)	7 (7–7)	0.207
Pathological staging				0.259
G1	57	43	14	
G2	1	0	1	
G3	0	0	0	
Delayed perforation: *n* (%)	0 (0%)	0 (0%)	0 (0%)	1.000
Delayed hemorrhage: *n* (%)	1 (1.72%)	1 (2.33%)	0 (0%)	0.746
Positive surgical margin: *n* (%)	4 (6.90%)	4 (9.30%)	0 (0%)	0.291
Infringement of blood vessels and nerves: *n* (%)	1 (1.72%)	1 (2.33%)	0 (0%)	0.746
Distance from tumor to the bottom margin < 500 um: *n* (%)	17 (31.03%)	17 (39.53%)	0 (0%)	0.002

M, median; QR, Quartile Range; ESD, endoscopic submucosal dissection; EFTR, endoscopic full-thickness resection.

## 4 Discussion

Most rectal NETs are no more than 10 mm (17, 18). Studies have shown that over 85% of patients are diagnosed at the G1 or G2 stage (18, 19). In this study, 52 (98.11%) patients were in the G1 stage, and 1 (0.89%) patient was in the G2 stage. This result is consistent with the aforementioned study. Although rectal NETs smaller than 10 mm have a low risk of metastasis, it is still recommended to perform resection. For rectal NETs smaller than 10 mm, endoscopic resection is the most appropriate method (20). Studies have shown that the endoscopic treatment methods for rectal NETs include endoscopic mucosal resection (EMR), band-assisted EMR, cap-assisted EMR, or endoscopic mucosal dissection (ESD) (20, 21). However, all of the above methods have the problem of positive margins. EFTR can completely remove the lesion without worrying about positive margins. However, the wound is larger, there is a risk of delayed bleeding and delayed perforation after the operation. Therefore, we conducted this prospective case-control study to evaluate the differences in postoperative complications and therapeutic efficacy between EFTR combined with nylon thread titanium clip closure of the wound and traditional ESD treatment.

In this study, there were no patients with postoperative delayed perforation in either the EFTR group or the ESD group. Among them, one patient in the ESD group experienced delayed bleeding, while all 15 patients in the EFTR group did not have delayed bleeding. That is to say, among the 15 patients in the EFTR group, no bleeding or perforation complications occurred after the operation. Moreover, compared with the ESD group, there was no significant difference in the length of hospital stay. This proves that EFTR is also a safe endoscopic treatment method for rectal NETs, and it does not increase the length of hospital stay for patients. The resection margins of patients in the ETFR group were all negative and all underwent curative resection. However, in the ESD group, the resection margins were positive for 4 patients. Although the difference was not statistically significant, it cannot be ruled out that this was due to the small number of patients in the EFTR group. Not only that, in the ESD group, 17 patients had a tumor distance from the bottom surgical margin of less than 500 micrometers, while in the EFTR group, all 15 patients had not this situation, and the difference was statistically significant. This demonstrates that EFTR tended to be associated with a lower rate of positive resection margins compared with ESD, which may help reduce the need for additional treatment and potentially improve patient outcomes. A retrospective study published in 2020 showed that the complete resection rate of EFTR was 100%, and there were no major adverse reactions (12). The results of this study are consistent with theirs.

In this study, endoscopic purse-string sutures with titanium clips and endoloops were used to close the surgical wound after EFTR. Place the nylon rope at the edge of the wound, attach several titanium clips, and achieve complete closure of the wound by retracting the nylon rope. By completely closing the wound, it avoids the occurrence of delayed postoperative perforation and bleeding. Previous study (22) has reported that this method even can close the duodenal perforation caused by ERCP.

This study has some limitations. Firstly, due to the large size of the EFTR wound, some patients randomly assigned to the EFTR group refused to undergo this treatment method, resulting in only 15 patients in the EFTR group, which is too small a sample size and reduces the credibility of the results. Secondly, this study did not observe the recurrence situation after the operation. Finally, this study is a single-center prospective study. Therefore, we hope that in the future, more multi-center large-sample studies will be conducted to evaluate the efficacy and safety of EFTR in the treatment of rectal NETs.

## 5 Conclusion

In conclusion, EFTR combined with endoscopic purse-string sutures is an endoscopic treatment method for rectal NETs that is safe and may tend to be associated with a lower rate of positive resection margins compared with ESD.

## Data Availability

The raw data supporting the conclusions of this article will be made available by the authors, without undue reservation.
